# Association between Laryngeal Airway Aperture and the Discharge Rates of Genioglossus Motor Units

**DOI:** 10.3389/fphys.2017.00027

**Published:** 2017-01-25

**Authors:** Amy LaCross, Peter J. Watson, E. Fiona Bailey

**Affiliations:** ^1^Department of Physiology, College of Medicine, University of ArizonaTucson, AZ, USA; ^2^Department of Speech, Language, and Hearing Science, College of Liberal Arts, University of MinnesotaMinneapolis, MN, USA

**Keywords:** genioglossus, motor unit, phonation

## Abstract

We know very little about how muscles and motor units in one region of the upper airway are impacted by adjustments in an adjacent airway region. In this case, the focus is on regulation of the *expiratory* airstream by the larynx and how changes in laryngeal aperture impact muscle motor unit activities downstream in the pharynx. We selected sound production as a framework for study as it requires (i) sustained expiratory airflow, (ii) laryngeal airway regulation for production of whisper and voice, and (iii) pharyngeal airway regulation for production of different vowel sounds. We used these features as the means of manipulating expiratory airflow, pharyngeal, and laryngeal airway opening to compare the effect of each on the activation of genioglossus (GG) muscle motor units in the pharynx. We show that some GG muscle motor units (a) discharge stably on expiration associated with production of vowel sounds, (b) are exquisitely sensitive to subtle alterations in laryngeal airflow, and (c) discharge at higher firing rates in high flow vs. low flow conditions even when producing the same vowel sound. Our results reveal subtle changes in GG motor unit discharge rates that correlate with changes imposed at the larynx, and which may contribute to the regulation of the *expiratory* airstream.

## Introduction

Human tongue muscles participate in respiration-related and voluntary movements. In regard to respiration, it is evident that the extrinsic tongue protrudor muscle genioglossus (GG) defends the airway against inspiratory narrowing at rest (Cheng et al., 2011), in exercise (Walls et al., 2013), and during sleep (Chuang et al., 2009). Much less is known of the GG's role in regulating airway lumen in activities that depend on control of expiratory airflow including coughing, speaking, wind instrument playing, and singing.

Sustained expirations that are the hallmark of conversational speech result from passive and active forces that operate on the chest wall (ribcage + abdomen; Grimby et al., 1968; Bunn and Mead, 1971; Agostoni et al., 1979) and active regulation of downstream resistances (Remmers and Bartlett, 1977; England et al., 1982; Giering and Daubenspeck, 1990). Indeed, for sound generating behaviors, structures such as the larynx (Finnegan et al., 2000; Gillespie et al., 2015), nose (Peters and Boves, 1988; Sapienza et al., 1997), and velopharynx (Warren, 1986; Warren et al., 1989) fulfill dual functions serving as variable resistors that regulate air pressure and airflow *and* as sites where the vowels and consonants of language are formed.

**Figure 1:**
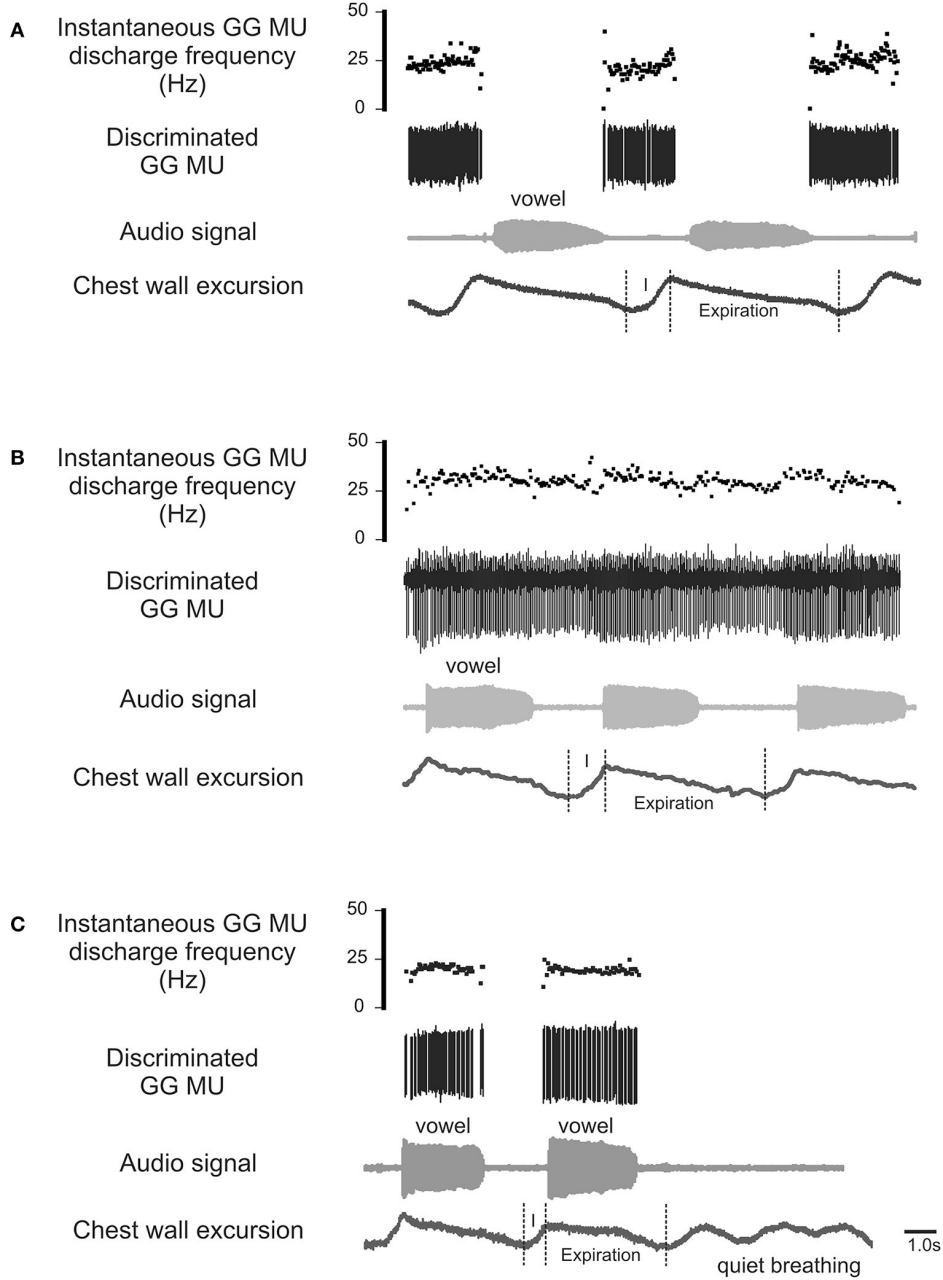
**Representative EMG recordings of GG single motor unit activities obtained from three different GG motor units**. Topmost trace in each panel shows the instantaneous motor unit discharge rate, middle trace the discriminated GG EMG motor unit, the audio signal and lower most trace depicts the chest wall motion. **(A)** Shows a GG motor unit active on inspiratory phase (I) of the breath cycle but silent during phonation on expiration. **(B)** is an example of a GG motor unit that exhibits tonic activation throughout the both the inspiratory (I) and expiratory phases of the breath cycle with some evidence of firing rate modulation during phonation. **(C)** Shows a GG motor unit active during phonation on expiration but silent on inspiration (I) and inactive during rest breathing. The activity patterns highlighted in **(C)** are the focus of the current study.

The process by which airway resistors are controlled and coordinated is of fundamental interest and importance both to speech and to respiratory motor control. Whereas, most previous research has examined the GG's role defending the airway on inspiration (Remmers et al., 1978; Mezzanotte et al., 1992, 1996; Fogel et al., 2001; Remmers, 2001), in this case we look for evidence of its *expiration-related* activity. Recently we documented expiration-related GG activity during moderate and heavy exercise that suggested a role for the muscle in the regulation of the expiratory airstream (Walls et al., 2013). In that circumstance, we hypothesized expiration-related GG activity is modulated in parallel with laryngeal airway aperture (England and Bartlett, 1982) to dilate the airway to reduce expiratory time. Here we explore the possibility further asking how changes imposed at the larynx in the process of speech communication, affect the downstream activation of genioglossus muscle motor units. Accordingly, we exclude from our analysis inspiratory motor unit activity (Figure 1A) and sustained or tonic motor unit activity (Figure 1B), electing to focus in this case on GG motor unit activity associated exclusively with the production of sound on expiration (Figure 1C).

## Methods

### Participants

Thirty-one adults (21 females and 10 males; mean age ± SD, 20–24 years) participated in the study. Only healthy participants (BMI ± SD, 22.69 ± 3.17 kg·m^2^) were recruited to the study. Adults who reported a history of respiratory disease or impairment, major surgery, or injury involving the upper airway or respiratory or sound production systems were excluded. All were native speakers of American English. The Human Subjects Committee at the University of Arizona approved all experimental procedures and subjects provided written, informed consent prior to participation.

### Experimental conditions

#### Laryngeal airway manipulations

In this study, subjects were required to produce two forms of sound energy (a) voice (phonation) and (b) whisper. In a third condition, subjects used an artificial sound source or electro-larynx. An explanation of each of these conditions and the rationale for their inclusion is provided below. The reader is directed to the experimental schematic provided in Figure 3 and the accompanying legend.

1. *Phonation*. Voice is produced when expiratory airflow from the lungs sets the vocal folds into vibration converting aerodynamic power into sound energy (e.g., acoustic power; Simonyan and Horwitz, 2011). Subjects were instructed to speak/produce sound at their normal conversational loudness (Seashore, 1938). In this condition, vocal folds approximate and create a resistance to the expiratory airstream that gives rise to a complex *periodic* sound energy.
2. *Whisper*. Subjects were instructed to imagine whispering in someone's ear. In this condition, vocal folds approximate the midline forming a glottis that is V or Y shaped (Solomon et al., 1989; Sundberg et al., 2010) that creates turbulent airflow and complex *aperiodic* sound energy (Monoson and Zemlin, 1984; Solomon et al., 1989; Matsuda and Kasuya, 1999). Expiratory airflow in this condition is higher and more turbulent than for phonation (Schwartz, 1968; Weismer and Longstreth, 1980; Stathopoulos et al., 1991).
3. *Electro-larynx condition*. This condition served as a control condition. The electro-larynx is a battery-powered device coupled to a small tube which, when placed in the corner of the mouth, introduces mechanically generated complex and *periodic* sound energy into the vocal tract. Because the EL is the sound source there is no requirement for vocal cord adduction or vibration as is the case for whisper and voice. Rather, subjects continue to breathe normally without interrupting sound production. Expiratory airflow is at its nadir in this condition.

#### Pharyngeal airway manipulations

**Figure 2:**
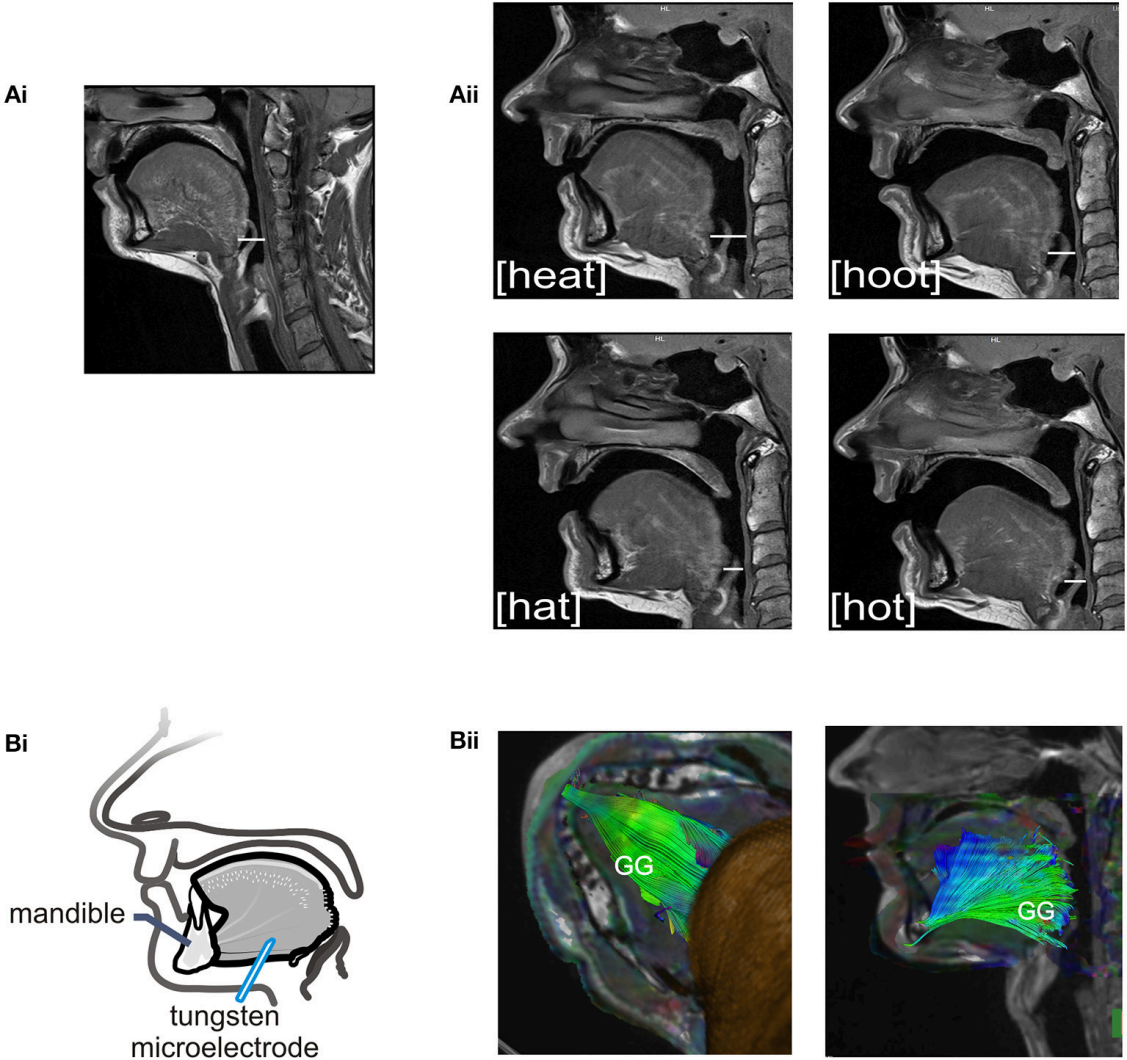
**(A)** Sagittal MRIs obtained from one subject during rest breathing and production of vowel sounds. **(Ai)** At left, the position of the tongue and the pharyngeal and oral airway lumens in quiet breathing. **(Aii)** Shows pharyngeal and oral airway lumens in production each of the vowels /i/, /æ/, /u/, /ɑ/ as heard in the words /heat/, /hat/, /hoot/, and /hot/. Note that the dimensions of the pharyngeal airway lumen (horizontal white line in each panel) are determined by the position of the tongue which differs somewhat for each vowel. The pharyngeal airway lumen is greater for the vowels in /heat/ and /hoot/ relative to /hat/ and /hot/ and relative to quiet breathing. **(Bi)** Schematic of the experimental set up showing approximate location of the recording electrode within the horizontal compartment of the GG. **(Bii)** A representative DT image from a subject showing muscle fiber tracts superimposed on MR images (low *B*-value magnitude images). This inferior view of the floor of mouth reveals the horizontal course of GG muscle fibers. EMG recordings were made within the GG muscle ~1.0–3.0 cm posterior to the mandible in muscle fibers that extend in a horizontal plane from the mental symphysis of the mandible anteriorly to the tongue base, in the region of the epiglottis, posteriorly. Activation of this compartment of the GG results in forward motion of the tongue and increased pharyngeal airway lumen.

The sound energy produced at the larynx is filtered and amplified as it passes through the upper airway. The sound produced by the larynx resonates in the chambers formed by the pharynx, nasal and oral cavities creating the sounds we recognize as vowels. Importantly, the dimensions of the pharyngeal airway are determined in large part by the tongue and changes in tongue placement are required to achieve each different vowel and to shift from vowel production to resting breathing (see Figures 2Ai–ii). Thus, each vowel is the result of a distinct pharyngeal airway geometry. We asked participants to say the vowels /i/, /æ/, /u/, /ɑ/ as pronounced in the words; *heat*, *hat*, *hoot* and *hot* (Figure 2Aii). Subjects sustained each vowel for 1–2 s and completed 6–8 repetitions per trial. There were no time constraints for initiation or completion of the task.

**Figure 3:**
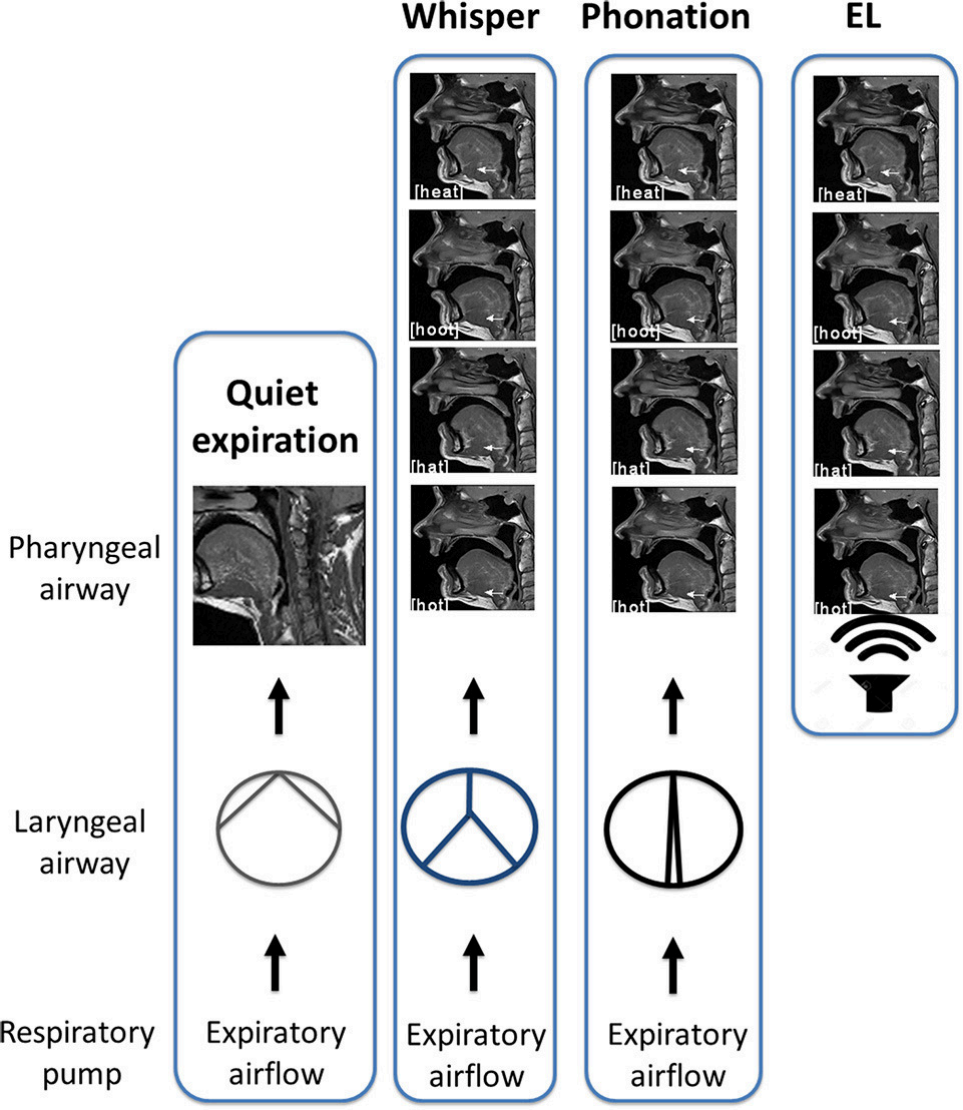
**Schematic of experimental conditions**. In quiet breathing, air exits the lungs via the laryngeal airway where vocal cord adduction/abduction controls airway lumen and therefore, airflow. The laryngeal airway is widest for quiet expiration. In whisper, the vocal folds approximate the midline creating air turbulence. Turbulent airflow and vocal cord vibration result in complex *aperiodic* sound energy that is characteristic of whisper. When the vocal folds are more closely approximated, resistance to the expiratory airstream increases and results in a complex *periodic* sound energy that is characteristic of phonation. Shown at right is the “electro-larynx” condition. As the name suggests, the electro-larynx provides a battery-powered sound source can be introduced into the airway via a small tube placed in the corner of the mouth. In this condition, the electro-larynx *not the larynx* energizes the upper airway creating complex periodic sound energy for speech. As depicted, whereas whispered (top most panel), phonated (second panel) and electrolarynx productions of a vowel share the same tongue shape and position within the oral cavity the laryngeal configuration is distinct to that condition.

Audio signals were recorded with the highest digital sampling rate available when multiplexed with the digitized physiologic (motor unit) data. The audio signal was recorded (16.667 kHz sampling frequency; 16 bit depth) via head-mounted microphone (Opus 55.18 MK II, BeyerDynamic, Long Branch, NJ) positioned 2 mm from the left corner of the mouth. To eliminate aliasing effects, the speech signal was filtered using a 10th-order variable low-pass filter unit (32 kHz, 4301, CED, Cambridge, UK) before transmission to a data acquisition interface (Power1401, CED, Cambridge, UK). F1 and F2 formant frequencies were identified for each utterance by visual examination of the spectrographic display in Praat (Version 5.3.14) and determined to be comparable to previously reported values in men and women (Peterson and Barney, 1952; Hillenbrand et al., 1995).

Chest wall motions were monitored via strain-gauge transducers (Pneumotrace, UFI, Morro Bay, CA) positioned around the thorax at the mid-sternal level and around the abdomen at the level of the umbilicus. Output from these sensors reflected changes in circumference of the rib cage and abdomen and were used to distinguish inspiration and expiration.

### Magnetic resonance imaging (MRI)

In this case, we obtained images of the upper airway in four subjects (two male and two female) Representative images obtained from one subject are presented in Figure 2. We include these images to make clear the configuration of the pharyngeal airway in the production of *of* /i/, /æ/, /u/, /ɑ/. Images were obtained using a Siemens Magnetom Skyra 3 Tesla MRI Scanner (University of Arizona, MRI Facility) and subjects lay supine in the scanner and received instructions from the experimenter via headphones prior to each production. Note that audio and motor unit recordings were not attempted during imaging sequences.

Sagittal, axial and coronal planes were imaged (on all planes: TE: 10 ms, TR: 1500 ms, ETL: 5 ms, FOV: 150 cm, Flip angle: 160°). For all scan types, an anterior neck coil (four element Flex coil, München, Germany) was used so that the desired portion of the head and neck were brought into the scanning field of view. To image GG muscle fibers for each vowel, a series of fifteen 3-mm thick contiguous, parallel, sagittal sections were gathered in an interleaved acquisition. The image set extended from just above the sinus cavities to the inferior border of the mandible.

#### Probabilistic diffusion tractography

We performed this additional analysis of imaged tissues to identify muscle fiber course. Tractography detects the direction of water diffusion and generates tracts across voxels yielding information about muscle fiber orientation (Gilbert et al., 2006). Tracts were extended and connected across voxels only when they met an angular threshold criterion <30° (Siemens Neuro 3D software, University of Arizona). In this case we used tractography solely to highlight the orientation and trajectory (Gilbert et al., 2006) of those GG muscle fibers in the posterior tongue that contribute to airway dilation (Miyawaki et al., 1975; Buchaillard et al., 2009; Cheng et al., 2011). Example images obtained using diffusion tractography in the same subject are presented in Figure 2Bii.

#### Electromyographic (EMG) activity

Motor unit recordings were obtained from the horizontal compartment of the GG using single tungsten microelectrodes (Frederick Haer, Bowdoinham, ME, 1–5 μm tip diameter, 10 MΩ at 1 KHz). Electrodes were inserted through the skin under the jaw into the mid-region of the GG muscle with entry points ~1.5 cm from the midline and ~2 cm posterior to the mandible (refer to Figure 2Bi). Tungsten electrodes cause little discomfort to the participant and do not require anesthetic of any kind. Depth of insertion was determined in advance via ultrasound (Pro Sound 3500, Aloka Co., Ltd., Tokyo, Japan). The depth from the skin surface to the GG varies with subject size and the depth to the inferior border of the muscle ranged from 1.5 to 2.5 cm from the skin surface (subjects BMI ± SD, 22.69 ± 3.17 kg·m^2^). A surface electrode (4.0 mm diameter Ag-AgCl) attached to the skin overlying the mastoid process served as the indifferent electrode and subjects were grounded via a 3 M Red Dot electrode (Ag-AgCl) affixed to the skin overlying the clavicle. Motor unit action potentials were amplified (x1000), band-pass filtered (0.3–3 KHz; Grass Instruments, West Warwick, RI), and displayed on a storage oscilloscope to monitor size and shape of the impulses during data acquisition. Single motor unit activities were recorded using Spike2 data acquisition software (CED, Cambridge, UK).

### Data analysis

Analysis of motor unit action potentials was performed offline in Spike2 (CED, Cambridge, UK). Action potentials were discriminated using a template-matching algorithm based on waveform shape and amplitude and subsequently checked by visual inspection against the template unit waveform as discussed previously (Bailey et al., 2007). The mean instantaneous discharge rate was determined in the interval between utterance onset and offset, defined by the first and final zero-crossings of the audio waveform (refer Figure 1C). In determining average discharge rate and variability, only motor units for which activities could be followed throughout a series of vowel productions were included. For each recorded motor unit, average discharge rates were calculated from the average of three trials (each trial comprising 10–12 repetitions) of each vowel.

Statistical analyses were performed using SAS software (version 9.3). We used a linear-mixed ANOVA model to assess the effects of sex, laryngeal airway condition (phonation vs. whisper) and pharyngeal airway condition (each of four vowels) on motor unit discharge rates. Laryngeal and pharyngeal conditions were coded as fixed effects, subject and motor-unit (within subject) were coded as random effects. Note that of the total number of motor units recorded in whisper and phonation (*n* = 116), only 12/116 were also recorded in the EL condition. Accordingly, the data from the EL condition were not included in the statistical analysis.

In the event of a significant *F*-value, differences were tested using *post hoc* comparisons with significance levels adjusted according to the Bonferroni procedure (*p* = 0.004). To analyze equality of variance of mean motor unit firing rates between vowel sounds, Levene's test of homogeneity was used to identify the variance of an individual motor unit's firing rate from the average firing rate for each vowel. Average firing rate was the dependent variable and vowel was the independent variable. Corrections for multiple comparisons were made using the Bonferroni procedure (*p* = 0.02).

## Results

We recorded the activities of 116 motor units in the region of the GG muscle that regulates the pharyngeal airway immediately downstream to the larynx in phonated and whispered productions of four vowels as follows; [i], [u], [æ], and [ɑ]. The average number of motor units obtained from female (4.1 ± 4.2) and male participants (3.1 ± 2.09) was not different (Mann-Whitney *U* = 109.0, *p* = 0.984). The challenges of obtaining single motor unit recordings during speech sound production precluded us from obtaining equal numbers of recordings from all subjects however, the total number of motor units recorded for each vowel in whisper and phonation was comparable: [i]: *n* = 26, [u]: *n* = 34, [æ]: *n* = 29, and [ɑ]: *n* = 27.

**Figure 4:**
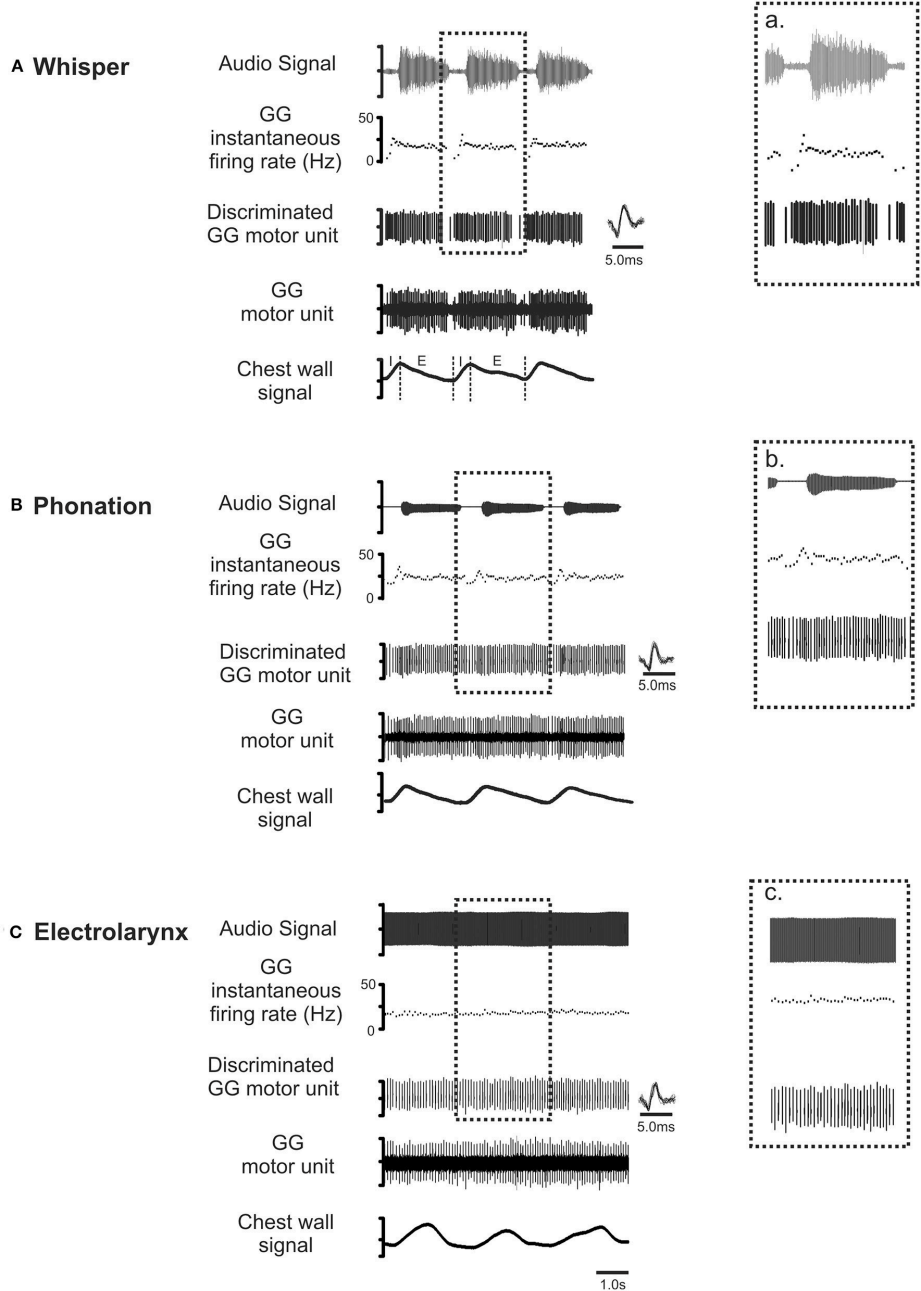
**Representative recordings of GG motor unit activity obtained from one subject in whisper (A)**, phonation **(B)** and electro-larynx **(C)** productions of the vowel /i/ as in /heat/. Topmost trace audio signal. Note that the greater amplitude audio signal in whisper **(A)** is the result of doubling the amplification in this condition relative to amplification settings for phonation and electro-larynx required to detect the speech signal in this condition. The middle three traces in each panel in show the untreated GG EMG signal, the discriminated GG single motor unit and the instantaneous motor unit discharge rate in each condition. Note that motor unit discharge averages in each task were determined in the expiratory (E) phase of each breath cycle in the interval between utterance onset and offset, defined by the first and final zero-crossings of the audio waveform. Note also, subtle differences in GG activity evident across the three conditions. Because tongue shape and position are presumed to remain stable to attain the target vowel, any between task differences in motor unit activity patterns are attributed to differences in valving of the expiratory airstream by the larynx that are characteristic of whisper, phonation and EL conditions (see Figure 3).

Figure 4 shows representative recordings of the activities of a motor unit during production of the same vowel in each of the three conditions: (A) whisper, (B) phonation, and (C) electrolarynx. At right, time expanded views highlight subtle differences in motor unit activity between whispered, phonated, and electro-larynx productions of the vowel. Whereas, whispered and phonated vowels were characterized by distinct patterns of activation, motor unit activity in the EL production of the same vowel shows no firing rate modulation.

There was a significant main effect of vocal condition [*F*_(1, 168)_ = 4.52, *p* = 0.035] with average motor unit discharge rates in whisper exceeding those in phonation. Interestingly, an interaction between sex and vocal condition approached significance [*F*_(1, 168)_ = 3.59, *p* = 0.06]. That is, whisper was associated with higher average discharge rates in men than in women. However, in the absence of a significant effect the data were collapsed across male and female participants. For the group as a whole, motor unit discharge rates ranged from a minimum of 10.3 Hz to a maximum of 25.8 Hz. Motor unit firing rates in phonation were below 20 Hz for the majority (26/31) of subjects whereas firing rates in whisper exceeded 20 Hz in 17/31 subjects. In contrast, the lowest average firing rates were recorded in the electro-larynx condition with firing rates ranging between 12 and 14 Hz.

**Figure 5:**
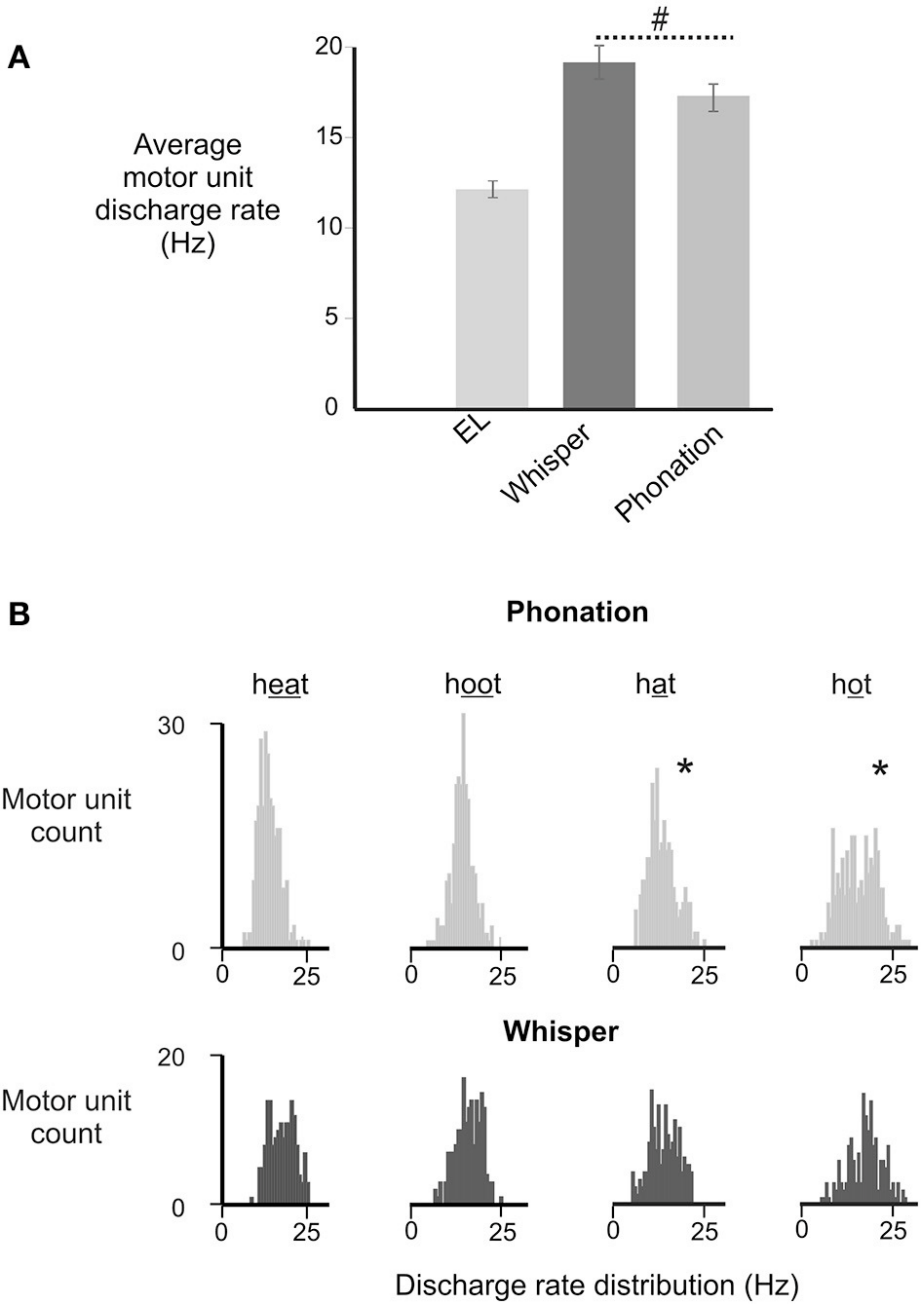
**(A)** Depicts the average (±SD) discharge rates for GG motor units for in electrolarynx, whisper and phonation conditions. (#) Indicates a significant between group difference (*p* < 0.05) in MU discharge rates between whisper and phonation. **(B)**. Depicts the distribution of MU discharge rates in whispered and phonated versions of each vowel. Significantly greater variability (indicated by asterisk^*^) in discharge rates was noted for vowels produced with more open jaw positions /æ/, /ɑ/ and for which there is little or no contact made between the tongue and maxilla and/or upper dentition and presumably therefore, less stability.

The distribution of motor unit firing rates for all subjects in whisper and phonation are presented in Figure 5B. Although average motor unit discharge rate for /i/, /æ/, /u/, and /ɑ/ were not different [*F*_(3, 95)_ = 0.82, *p* = 0.448], the discharge rate variances were different [*F*_(3, 79)_ = 2.364, *p* < 0.03]. Specifically, the average firing rate was more variable for vowels produced with a more open mouth and lower tongue position ([æ] and [ɑ]) (SD: 3.97 Hz) relative to than vowels produced with a more closed mouth and correspondingly higher tongue position [(i) and (u) (SD: 2.79 Hz) [*F*_(1, 50)_ = 7.257, *p* < 0.01].

## Discussion

This study has three main findings. First, we provide evidence that the posterior region of the GG may contribute to changes in airway shape and stiffness in speech tasks that are performed exclusively *on expiration*. Second, we show that motor unit activity patterns in the EL condition are distinct from those in whisper and phonation. Thus, whereas motor unit activity persists throughout EL productions of each vowel, average motor unit firing rates decline and flow dependent modulation is eliminated. Last, in the switch from a low airflow condition (phonation) to a higher airflow condition (whisper), GG motor unit firing rates increase consistent with an increase in muscle activation. Given the GG's origin on the mandible, such activation likely results in forward motion of the tongue base and to *expiration-related* airway dilation.

### Experimental method

Although GG activity on expiration has been characterized previously (Sauerland and Mitchell, 1970; Sauerland and Harper, 1976; Saboisky et al., 2006, 2007) this aspect of the muscle's function has garnered much less attention. Recently, we recorded GG motor unit activity during moderate and heavy bicycling and noted that GG activation persisted throughout both the inspiratory and expiratory phases of each breath cycle (Walls et al., 2013). In light of the aforementioned, we sought alternative behavioral contexts in which to assess expiration-related GG activation. Speech, and sound production more broadly, is ideal in this regard because it entails volitional control of respiration (Loucks et al., 2007) and requires sustained expirations and because laryngeal and pharyngeal airway muscles participate in the regulation of the expiratory airstream. Using this approach, we were able to effect changes in laryngeal airway aperture simply by asking subjects to produce whispered or phonated versions of four vowels. We incorporated a third condition, the electrolarynx condition, to uncouple pharyngeal, and laryngeal airways while still operating within the framework of sound production. These manipulations provided a means of gauging how expiration-related GG activity correlates with change/s imposed at the larynx.

### Regulation of expiratory airflow

Previously published airflow rates for whisper encompass the range 0.2–0.9 L/s (Monoson and Zemlin, 1984; Stathopoulos et al., 1991; Sundberg et al., 2010) and for phonation, 0.08–0.17 L/s (Terasawa et al., 1987; Bailey and Hoit, 2002). Interestingly, these values are higher (whisper) and lower (phonation) than air flow rates reported during moderate-heavy exercise 0.25–0.43 L/s (Walls et al., 2013). However, a key distinction between speech/sound production and exercise lies in the lung volume excursions for the two tasks. In speech or sound production, lung volumes excursions typically encompass ~400–500 ml (Bailey and Hoit, 2002) as compared to ~850 ml in moderate and heavy exercise (Walls et al., 2013). This distinction is an important consideration because GG EMG is modulated by feedback from pulmonary stretch receptors (PSRs) (Brouillette and Thach, 1980; van Lunteren et al., 1984). PSRs are stimulated by lung inflation but in adults, the volume threshold for their activation is ~1.5–2.0 times the individual's resting tidal volume (Lind and Hesser, 1984). Whereas, tidal volume excursions of this magnitude are more common in heavy exercise and likely contribute to PSR-related inhibition of GG EMG, conversational speech operates within the mid-range of lung volumes and thus, it is unlikely that PSR feedback is triggered in this context.

### Motor unit discharge

Although there were no differences in motor unit firing rates between vowels, discharge rates varied more in the production of low and back vowels than in high and front vowels (Figure 5B). Differences in discharge rate variability previously have been attributed to differences in nervous system control—specifically the number of inputs that converge onto the motoneuron however, for the tongue this variability more likely is a function of the position of the jaw upon which the tongue rests (Shiller et al., 2002; Iskarous et al., 2011). As noted, GG motor unit discharge variability was lowest in production of the vowels /i/, and /u/ (i.e., heat and hoot) that are produced with a relatively closed mouth and with contact made between the tongue and the palate and/or teeth. Conversely, discharge variability was greatest for the vowels /æ/, /ɑ/ (i.e., hat and hot) that are produced with a more open mouth and with no contact made between the tongue and palate and/or teeth. Thus, in the absence of a bony skeleton, the tongue's contact with an external bony target may confer much-needed stability (Gick et al., 2013).

Although we observed a trend toward somewhat higher average motor unit discharge rates in men than women, there is very little data that supports the notion of sex–related differences in motor unit activation patterns. Whereas, several previous studies point to sex based differences in fatigability (Semmler et al., 1999; Bilodeau et al., 2001; Hunter et al., 2006), evidence of sex-based differences in motor unit firing rates are harder to find. To our knowledge, only one previous study (Christie and Kamen, 2010) noted differences in maximal motor unit discharges rates that were ~9.0% higher in young men than in young women (32.7 ± 6.8 Hz vs. 29.3 ± 7.0 Hz, *p* = 0.05). Nevertheless, in view of the unequal number of male (*N* = 10) and female (*N* = 21) participants in the current study, the possibility remains that the difference reported here is a function of non-physiological factors (Hunter, 2014).

### Summary

We show increases in GG activation that occur against a background of changing airflow through the glottis. These findings are consistent with the notion of complimentary regulation of the laryngeal and pharyngeal airways (McClean and Tasko, 2002) and of the system wide regulation of airflow (Warren, 1986) but differ from previously published findings that show a diminution in expiration-related GG activities during moderately heavy exercise (Walls et al., 2013).

## Author contributions

AL: acquisition of EMG data, analysis and interpretation of data and approval of the submitted version. PW: conception and design, interpretation of data, writing of the manuscript and approval of the submitted version to be published. EB: conception and design, interpretation of data, reporting of the results, writing of the manuscript and final approval of the version to be published.

### Conflict of interest statement

The authors declare that the research was conducted in the absence of any commercial or financial relationships that could be construed as a potential conflict of interest.
